# Gold Nanostructures as a Platform for Combinational Therapy in Future Cancer Therapeutics

**DOI:** 10.3390/cancers3011081

**Published:** 2011-03-04

**Authors:** Salomeh Jelveh, Devika B. Chithrani

**Affiliations:** 1 Ontario Cancer Institute, Princess Margaret Hospital, University Health Network, Toronto, ON, Canada; E-Mail: sjelveh@uhnres.utoronto.ca; 2 Department of Radiation Physics, Princess Margaret Hospital, Toronto, ON, Canada; 3 STTARR Innovation Centre, Toronto Medical Discovery Tower, Toronto, ON, Canada

**Keywords:** gold nanostructures, drug delivery, radiation therapy, photothermal therapy, photodynamic therapy, combinational therapy

## Abstract

The field of nanotechnology is currently undergoing explosive development on many fronts. The technology is expected to generate innovations and play a critical role in cancer therapeutics. Among other nanoparticle (NP) systems, there has been tremendous progress made in the use of spherical gold NPs (GNPs), gold nanorods (GNRs), gold nanoshells (GNSs) and gold nanocages (GNCs) in cancer therapeutics. In treating cancer, radiation therapy and chemotherapy remain the most widely used treatment options and recent developments in cancer research show that the incorporation of gold nanostructures into these protocols has enhanced tumor cell killing. These nanostructures further provide strategies for better loading, targeting, and controlling the release of drugs to minimize the side effects of highly toxic anticancer drugs used in chemotherapy and photodynamic therapy. In addition, the heat generation capability of gold nanostructures upon exposure to UV or near infrared light is being used to damage tumor cells locally in photothermal therapy. Hence, gold nanostructures provide a versatile platform to integrate many therapeutic options leading to effective combinational therapy in the fight against cancer. In this review article, the recent progress in the development of gold-based NPs towards improved therapeutics will be discussed. A multifunctional platform based on gold nanostructures with targeting ligands, therapeutic molecules, and imaging contrast agents, holds an array of promising directions for cancer research.

## 1. Introduction

Surgery, radiation and chemotherapy remain the most widely used treatment options in the fight against cancer. Despite recent progress in conventional methods, there is still a great need for new treatments that can eradicate cancer cells while causing much less damage to the normal cells. As a step forward in this direction, researchers around the world are making great efforts to incorporate nanotechnology into existing therapeutics and imaging in cancer treatment. The flexibility of cancer nanotechnology allows the development of safer yet more effective diagnostic and therapeutic modalities for cancer therapy [1,2]. The ultimate goal of nanoparticle (NP)-based platforms will be the targeted delivery and monitoring of therapeutics to tumors while causing minimum side effects [3-7]. In addition, NP-based technology has the capability to develop novel multiplex systems to combine more than one treatment and imaging modality for creating a more aggressive and effective approach in eradicating cancer [8]. The first generation of NP-based therapies approved by the FDA consists of lipid based NP-systems, such as liposomes and micelles [9]. However, recently more attention has been given to develop inorganic NP-based systems, such as gold and magnetic NPs, for drug delivery and therapeutics [10].

**Scheme 1.**
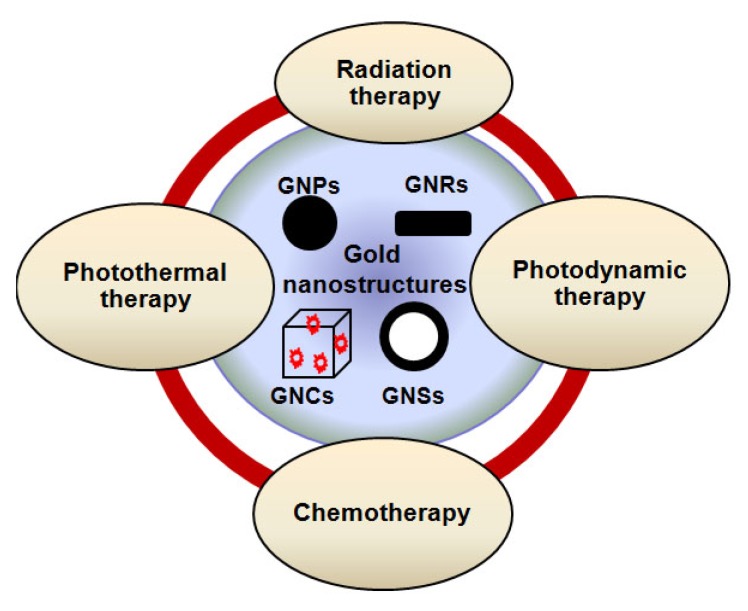
The possibility of combinational therapy for effective therapeutics in cancer treatment. Gold nanostructures are at the center of attention since they can be used as radiation sensitizers, anticancer drug enhancers, heat generators, and also effective drug carriers.

Among other inorganic NP-systems, gold nanostructures play a bigger role in cancer therapeutics because they: a) can enhance the damage induced by radiation and anticancer drugs; b) produce heat on exposure to UV and Near Infra Red (NIR) radiation and hence provide the possibility of destruction of cancer cells through thermal ablation; c) enhance the delivery of drugs such as anti-cancer drugs that are highly water-insoluble or unstable in the biological environment; d) increase the life time of drugs and imaging agents through NP-surface modification to avoid drug loss due to rapid clearance and metabolism [11,12]. Hence, gold nanostructures are being used as a therapeutic agent in radiation therapy (RT), chemotherapy, photothermal therapy (PTT), and photodynamic therapy (PDT). These new opportunities allow innovations leading to effective combinational therapy in the fight against cancer as illustrated in Scheme 1.

**Figure 1.**
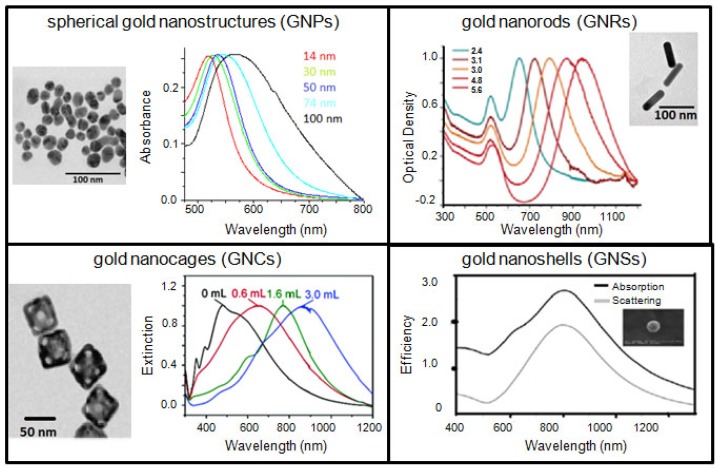
TEM images and optical properties of different sized and shaped gold nanostructures: spherical GNPs (top left), GNRs (top right), GNCs (bottom left), and GNS (bottom right), respectively. Reproduced with permission [13-16].

Gold-based therapeutic systems discussed in this article are spherical GNPs, GNRs, GNSs, and GNCs. Typical transmission electron microscopy (TEM) images and optical properties of the gold nanostructures discussed in this article are shown in Figure 1. The ultimate goal is to combine these features and build a better GNP-based platform, which may be potentially useful in a clinical setting. In addition, GNPs are also being used as a model NP-system to further optimize the interface between nanotechnology and medicine since they can be synthesized with different sizes, shapes, and surface properties as discussed in a recent review [17]. These NPs are also extensively being explored for applications in imaging techniques, such as, Computer Tomography (CT), Surface Enhanced Raman Scattering (SERS), and optical imaging [18-29]. However, in this review article, GNP-based therapeutic systems will be discussed as a promising NP-platform for combinational therapy for improved outcome in cancer therapy.

## 2. Gold Nanostructures as Sensitizers in Radiation Therapy

Radiotherapy remains a major modality of cancer therapy. A clear understanding of fundamental mechanisms of cancer biology and therapies can lead to improved clinical outcomes [30,31]. Understanding the fundamental mechanisms that induce DNA damage and cell death should lead to a clearer picture of the cause of cancers and benefit the development of improved strategies for cancer treatment. Radiation is used in radiotherapy since radiation (X-rays, γ-rays and fast-moving charged particles such as ions, electrons and protons) interacts with DNA inside living cells causing enough damage and that could lead to cell death [32]. For this reason, such radiation is used in radiotherapy to kill cancer cells.

**Figure 2.**
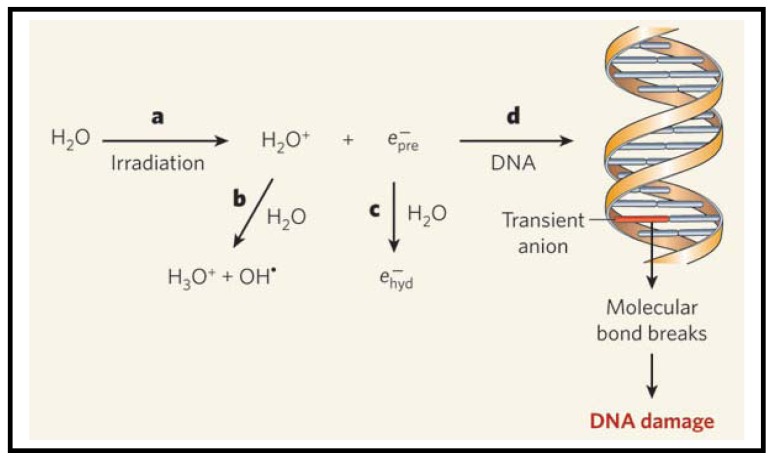
Mechanisms of radiation induced DNA damage. (**a**) Absorption of high-energy radiation by water molecules results in formation of H_2_O^+^ ions and free electrons. After losing their kinetic energy, the electrons enter a short-lived, prehydrated state $\left(e_{\mathrm{pre}}^{-}\right)$. (**b**) The H_2_O^+^ ions react with more water molecules to form protonated water (H_3_O^+^) and hydroxyl radicals (OH^•^). These radicals have long been thought to cause the DNA damage observed in irradiated cells. (**c**) Prehydrated electrons form complexes with water molecules and turn into hydrated electrons $\left(e_{\mathrm{hyd}}^{-}\right)$. (**d**) Prehydrated electrons also react with the bases of certain nucleotides in aqueous solution. This suggests that prehydrated electrons can react with the bases of DNA duplexes, forming transient anions. In some cases, these anions could decompose, breaking molecular bonds in the DNA and so damaging it. Reproduced with permission [33].

As illustrated in Figure 2, radiation produces ions, radicals and free electrons, as they travel through matter [32-34]. The electrons in turn generate large quantities of a second generation of radicals, ions and free electrons. Most studies suggest that DNA is damaged indirectly by hydroxyl radicals [35]. However, the electrons can also cause damage to DNA, as illustrated in a recent study in which low-energy electrons emitted from metal films were found to cause DNA strand breaks directly [36]. This study was performed using dry films. However, it is important to look at the role of electrons in a biologically relevant environment, such as water. When electrons are generated in water, they become hydrated and form a complex with several water molecules as illustrated in Figure 2. It was assumed that these hydrated electrons do not cause much DNA damage as compared to hydroxyl radicals [33]. Now, the question is whether these hydrated electrons can cause DNA damage. Recently, Wang *et al.* performed an experiment to study the reaction of prehydrated electrons with deoxyribonucleotides, the building blocks of DNA [37]. The authors performed their experiments in water, which provides a good model for cells. They found that significant quantities of single- and double- strand breaks of irradiated aqueous DNA are induced by prehydrated electrons. Based on these recent studies, both electrons and hydroxyl radicals could be responsible for DNA damage in irradiated cells. In the next section, we will discuss the contribution from GNPs to these existing mechanisms of cell damage after exposure to radiation.

**Figure 3.**
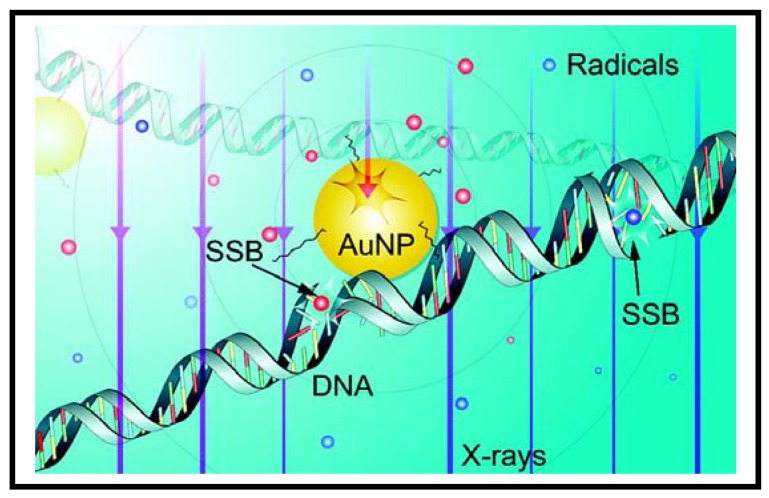
Mechanisms of radiation induced DNA damage in the presence of the GNPs: Schematic diagram of the results of a Monte Carlo simulation. Also shown are the radicals (blue spheres, distributed evenly) generated from electrons produced in water, as well as radicals (red spheres, concentrated near the GNP) from Auger electrons, secondary and photoelectrons originated from the GNP. The trajectories of electrons are not shown, and only the relative average density of radicals generated from these electrons is displayed. The diameter of the GNP shown here is approximately 3 nm. Reproduced with permission [38].

Recently, GNPs are being used as sensitizers in radiation therapy [39-44]. As a step forward towards understanding the mechanism behind enhanced sensitization properties of GNPs, Carter *et al.* have performed a Monte Carlo calculation and pointed out that the following effects can be combined to cause this phenomenon: (1) enhanced localized absorption of X-rays by nanostructures; (2) effective release of low-energy electrons from GNPs; and (3) efficient deposition of energy in water in the form of radicals and electrons. When GNPs are present, the electrons released from these NPs could create more radicals as illustrated in Figure 3. They also confirmed the theoretically predicted nanoscale energy deposition distribution by measuring hydroxyl radical-induced DNA strand breaks. These results provide important information towards understanding gold-based sensitization mechanisms. However, in these studies, the GNPs were in close proximity to DNA. The exact mechanisms of cell damage when GNPs are localized away from DNA (either when they are in the media or in the cytoplasm of the cell) are not known yet. Hence, more work needs to be done in order to elucidate mechanism of sensitization due to GNPs. There is great interest among many research groups to exploit the enhanced radiation sensitization property of GNPs to further improve radiation therapy as discussed below.

**Figure 4.**
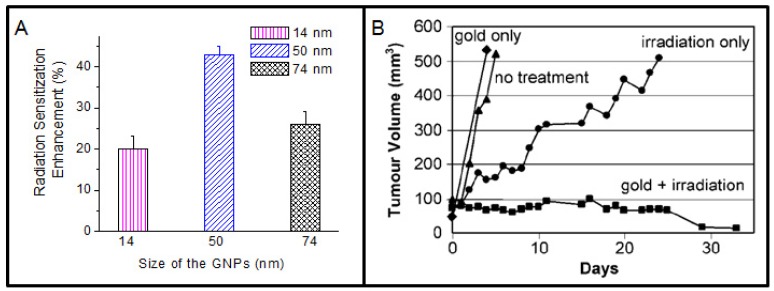
Radiation enhancement properties of GNPs. (**A**) Variation of cell survival fraction as a function of size of the GNPs (*i.e.*, 10% survival, 220 kVp). (**B**) Average tumor volume after: no treatment (triangles); gold only (diamonds); irradiation only (30 Gy, 250 kVp, circles); intravenous gold injection (1.35 g Au/kg) followed by irradiation (squares). Reproduced with permission [12,45].

An early study showed a dose enhancement effect for cells suspended in solutions with gold microspheres and also for tumors injected with gold microspheres [39]. In this case, microspheres could not penetrate the cells since their size was comparable to the size of the cells. In order to overcome this difficulty, GNPs of size range between 1-100 nm are now being used. Recent studies have shown that there is an enhancement in radiosensitization when GNPs are internalized in cancer cells [12,41,42,44]. The radiation enhancement factor was dependent on the size of the NPs, concentration of NPs, and cell type. Figure 4A shows the size dependent radiation response of GNPs. It is believed that the size of the NPs plays a big role in their uptake at the cellular level leading to different sensitization properties [12,16]. Using clinically relevant radiation sources, an enhancement in DNA double strand breaks (DSBs) was shown even 24 hrs after irradiation for cells with internalized NPs [12]. In this study, GNPs were localized in the cell cytoplasm and exact mechanisms of enhanced DNA damage, when GNPs are not in close proximity to DNA, is not yet fully known.

An important milestone in the field of radiation therapy was reached when Hainfeld *et al.* conducted the detailed experiments *in vivo* to explore the enhancement effect of GNPs and the data showed the potential utility of GNPs for cancer X-ray therapy [45,46]. They demonstrated that EMT-6 mammary tumors implanted in mice that received an intravenous injection of 1.35 g GNPs /kg could be completely eradicated in 30 days after irradiation with 250 kVp X rays as illustrated in Figure 4B [45]. However, detailed mechanisms leading to such impressive results are not yet known. It is believed that a larger portion of the energy of the primary ionizing photons is transferred to the tumor due to the increased absorption of X rays by GNPs [38,46-48]. In this experiment, mice were irradiated only 2 min after intravenous injection of GNPs, which is far too short to get a significant accumulation in the tumor cells. Recent *in vitro* studies suggest that NP-uptake is maximized hours after incubation and the enhancement of radiosensitization is dependent on the number of internalized NPs [12]. The study by Hainfeld *et al.* primarily focused on using low energy irradiations and the concentration of NPs used was high. Recently, Chang *et al.* demonstrated the feasibility of obtaining dose enhancement effects with a lower concentration of GNPs in tumor bearing mice in combination with clinical electron beams [49]. GNPs were injected intravenously into the mice and they used a GNP concentration of 1 g/kg compared to 2.7 g/kg by Hainfeld *et al.* [45,49]. Besides, they have reported higher accumulation within the tumor compared to tumor periphery, when the irradiation was done 24 hrs post-GNP injection (see supplement S1). This may be due to the enhanced permeability and retention effect, which takes advantage of the poorly formed tumor vasculature [50,51]. In future studies, GNPs can be surface modified for preferential targeting of the cancer cells [52]. If GNPs can be localized within the tumor, it would lead to a higher dose to the cancerous tissue compared with the dose received by normal tissue during a radiotherapy treatment.

A Monte Carlo study was done to estimate the tumor dose enhancement effects due to GNPs during a typical radiation treatment [53]. It was estimated that the dose enhancement for a 140 kVp X-ray over the tumor volume can be at least a factor of 2 at a concentration of 7 mg Au/g in the tumor assuming no gold outside the tumor. The assumption that NPs are localized only in the tumor is not true in practical applications involving mice. For example, several research groups have shown that the NPs were distributed not only in the tumor but also in the tumor periphery, muscle, liver, kidneys, and blood (see supplement S1) [45,49]. In order to fully understand the mechanism of sensitization due to GNPs, we need to look into the distribution of NPs within the tumor cells in those *in vivo* experiments to further conclude anticancer effects of GNPs during radiation treatments. As discussed earlier, primary target of radiation is nuclear DNA, with DSB formation causing the most lethal DNA damage. However, evidence is now emerging to suggest that radiation damage to mitochondria and the cell membrane may also contribute to the cytotoxic effect of radiation [54]. In addition, a recent study showed that GNPs were shown to potentiate the effect of the radiomimetic agent, bleomycin, in the absence of radiation, suggesting biological interactions of GNPs with cells could be another mechanism by which sensitization occurred [55]. If sensitization is primarily an effect of biological interactions with GNPs, the importance of GNP radical production, hypoxia, and cell signaling pathways needs to be elucidated. These findings would lead us to further future experiments in order to fully understand the mechanism of radiosensitization by GNPs. The physics of enhanced radiotherapy using gold is described in detail in a recent review article by Hainfeld *et al.* [56].

## 3. GNPs for Improved Drug Delivery and Enhanced Therapeutic Effects

Most current NP drug delivery systems are polymer or lipid-based [57-69]. The flexibility and ease of incorporation of drug molecules, imaging contrast agents, tumor-targeting ligands, and anticancer drugs into those NPs make them more promising for targeted therapy. Recently, more attention is being given to inorganic NPs such as GNPs as promising NP-platforms for improved drug delivery and therapeutics [10,11,70-75]. GNPs are an ideal drug-delivery scaffold because they are known to be nontoxic and nonimmunogenic [76,77]. GNPs can also be easily functionalized with targeting molecules and have shown excellent potential for the targeted delivery of drugs [78,79]. In addition, the possibility of incorporating GNPs into liposomes, micelles or dendrimers has increased the scope of future application of GNPs in drug delivery applications. [10,80-85].

Based on recent studies, it is clear that GNPs can be used for improved drug delivery [11,86-88]. For example, Visaria *et al.* have designed a GNP-based therapeutic system for the synergistic enhancement of hyperthermic cancer therapy by selective thermal sensitization and induction of vascular injury at the tumor site [87]. They used GNPs to selectively deliver an inflammatory cytokine, tumor necrosis factor alpha (TNF). Mice that were treated with TNF-conjugated GNPs had improved survival rates compared with mice that were treated with native TNF alone and the results are in agreement with their previous study [89]. In these drug delivery studies, polyethylene glycol (PEG) was added to NP-drug conjugate to avoid detection and clearance by reticuloendothelial system (RES) [71,87,90]. They have shown that this new vector altered the biodistribution and improved the drug safety and efficacy of drug treatment in tumor models. Recently, a Phase I study was completed by CytImmune to evaluate safety, pharmacokinetics, and efficacy of gold NP-drug conjugates (called “CYT-6091 or Aurimune”) [91]. According to the study, it was possible to safely and systemically deliver TNF in humans far beyond concentrations attained in previous human studies while the fever side effect was easily managed. In addition, tissue biopsies from treated patients showed that the NP-based drug accumulates in and around tumor sites, minimizing uptake by healthy tissues and immune system detection. Based on the success of the Phase I clinical trial, a Phase II clinical program is underway combining Aurimune with chemotherapy for treating pancreatic cancer, melanoma, soft tissue sarcoma, ovarian, and breast cancer patients.

Recently, it was shown that GNPs can be used to enhance DNA damage caused by platinum-based anticancer drugs and the enhancement effect of cisplatin by GNP was obtained when DNA was exposed to low energy electrons, as produced by ionizing radiation.[92]. These platinum-based anticancer drugs cisplatin, carboplatin, and oxaliplatin are an important component of chemotherapy and have had a major impact, particularly for patients with testicular or ovarian cancer [93]. As a proof of concept, Sanche and coworkers have shown that there is an enhancement in the DNA DSBs when anticancer drugs are used in combination with GNPs and ionizing radiation [92]. The GNP-DNA and DNA-cisplatin complex were prepared by mixing DNA or the DNA-cisplatin complex with GNP solution. They found that radiation-induced DNA DSBs, a highly lethal type of cellular damage, were enhanced by a factor of 7.5 by this combination.

In this study, GNPs were in close proximity to DNA. Hence, it would be interesting to carry out further experiments to see the full potential of these enhanced anticancer effects *in vitro* and *in vivo* where NPs are mostly localized in the cytoplasm away from DNA in the nucleus. As a step in this direction, Brown *et al.* have tethered the active component of the anticancer drug oxaliplatin to a PEGylated GNP for improved drug delivery and the *in vitro* study showed that drug-tethered NPs demonstrated as good as, or significantly better, cytoxicity than drug alone in cancer cell lines, such as lung epithelial cancer cell line and colon cancer cell lines (HCT 116, HCT15, HT129, and RKO ) [11]. The larger surface area of NPs facilitates attachment of a large number of drug molecules and they demonstrated that 280 drug molecules can be attached to a single NP. Based on these new findings, GNPs can be used for improved cancer therapeutics by combining chemotherapy and radiation for a better outcome in future cancer care of patients.

One of the main concerns in chemotherapy is the side effect of anticancer drugs due to their nonspecific attack on all rapidly dividing cells. GNPs can be used to resolve this limitation in chemotherapy through targeting and effective loading. Drug loaded NPs can be targeted to specific locations using either active or passive targeting strategies [94]. Drugs can be passively targeted to solid tumors through the enhanced permeability and retention effect [95]. Alternatively, drugs can be actively targeted to tumors through cancer-related receptors (such as folate, Epidermal Growth Factor (EGFR), transferrin) [96-99]. Once drugs are targeted to a specific location, it will be very important to control its release. There has been interesting research in progress to use gold nanostructures, such as, GNCs and GNRs for controlled release of drugs by using their photothermal effects [15,73,100-105]. Reports of the photothermal effect being used to facilitate the controlled release of therapeutic oligonucleotides from GNPs have only recently begun to appear [73,102-105]. Pulsed lasers are preferred in controlled release of drugs since the process occurs on the order of minutes rather than hours, which is typical for continuous wave (cw) lasers [73,103,106].

**Figure 5.**
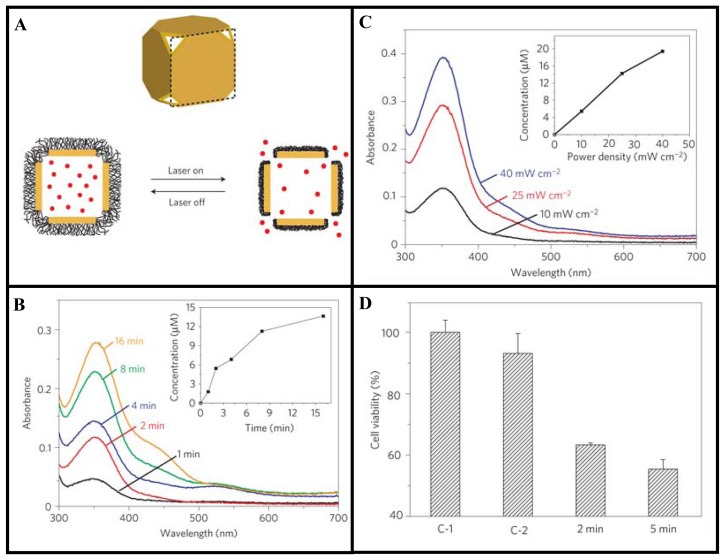
Use of gold nanocages (GNCs) for drug delivery. (**A**) Schematic illustrating how the system works. On exposure to a NIR laser, absorbed light is converted into heat, triggering the smart polymer to collapse and thus release the drug. When the laser is turned off, the polymer chains will relax back to the extended conformation and terminate the release. (**B**) Proof of concept: Absorption spectra of dye, dalizarin-PEG, released from the GNCs after exposure to a laser for different time intervals. (**C**) Controlled release of an anticancer drug, Dox, from the GNCs. (**D**) Cell viability after different treatments: (C-1) cells irradiated with a laser for 2 min in the absence of GNCs; (C-2) cells irradiated with the laser for 2 min in the presence of drug-free GNCs; and (2/5 min) cells irradiated with the laser for 2 and 5 min in the presence of drug-loaded GNCs. The power densities used for C-E are 40, 20, and 20 mWcm^−2^, respectively. The concentration of GNCs was 0.5 nM. Reproduced with permission [15].

Using a pulsed laser, Xia and coworkers have demonstrated that controlled release of drugs from GNCs was dependent on laser power and irradiation time [15]. These nanostructures have hollow interiors and porous walls [107,108]. The strong absorption in the NIR has been used to generate heat for the release of drugs. For example, the surface of a GNC can be covered with a smart polymer, and the pre-loaded drug can be released in a controllable fashion using a NIR laser as illustrated in Figure 5A [15]. They have demonstrated that drug release was dependent on both laser power and irradiation time (see Figure 5B-5C) [15]. They tested the technique with doxorubicin (Dox), a commercial chemotherapeutic anticancer drug for breast cancer and found that the drug release profile from the copolymer-covered GNCs was similar to what was observed for the dye. The same approach was then extended to an *in vitro* study that involved killing of breast cancer cells with the anticancer drug. This study shows the variation of cell viability depending on the laser irradiation time controlling the release of the drug (see Figure 5D). This is an example of a novel gold-based NP device that can be further improved to control the release of anticancer drugs in future chemotherapy. A recent review on the use of GNCs for drug delivery will provide additional information on this topic [100].

**Figure 6.**
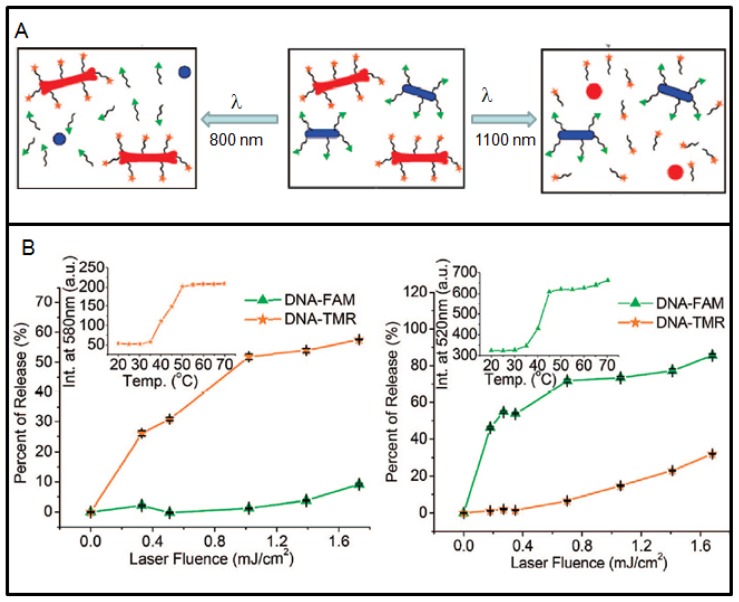
Use of gold nanorods (GNRs) for controlled delivery of drugs. (**A**) Schematic explaining the selective release. Laser irradiation of DNA-conjugated NRs (blue ovals) and NRs (red bones) are exposed to λ_800_ irradiation (left), which melts the nanocapsules and selectively releases the conjugated DNA (labeled by FAM (green triangles)). Exposure to λ_1100_ irradiation (right) melts the nanobones, selectively releasing the conjugated DNA (labeled by TMR (orange stars)). (**B**) DNA functionalization of NRs and selective release: Percent released of FAM-DNA (green triangles) and TMR-DNA (orange stars) as a function of λ_800_ and λ_1100_ laser fluence, respectively. Inset figures: melting curves of released DNA. Reproduced with permission [73].

Now, researchers are looking into ways of controlling delivery of more than one anticancer drug. Recently, GNRs are being used to demonstrate that it is possible to load and selectively release two different drugs from two different NRs by matching laser excitation wavelength to its infrared surface plasmon resonance (SPR), as illustrated in Figure 6A [73]. Two distinct DNA oligonucleotides were used to prove this concept (see Figure 6B). The specificity of DNA release was controllable externally. NRs have relatively large surface area and the capacity to load hundreds of molecules, and ∼80% of the payload can be released. In this case, pulsed laser irradiation was used for cleavage of the Au-S bond anchoring thiolated DNA to the particle's surface [73,103]. However, breaking the Au-S bond could also release free thiols causing detrimental effects in live cells. In order to overcome these limitations, new strategies have been used recently, such as, (a) utilizing doublestranded DNA_nanoparticle conjugates in which only one of the two oligonucleotide strands is anchored to the NP surface through a Au-S bond; (b) Irradiation of these systems with cw lasers triggered the photothermal effect and raised the local temperature above the melting temperature of the DNA duplex, which allowed the nonthiolated strand to dissociate into the surrounding medium while its complement remained attached to the NP [104,105]. The appeal of this process is primarily that the NP remains unchanged after the photothermal and release events. This approach, however, has its own limitations. It generates relatively low photorelease efficiencies and requires irradiation periods that is nearly twice as long as the examples using pulsed lasers in order to attain measurable degrees of oligonucleotide release. Both limitations are presumably attributable to the characteristically lower power densities attainable by irradiation with cw *versus* pulsed lasers. However, when pulsed laser are used, it can trigger melting of the NPs as shown in Figure 6A. Hence, it is important to consider the type of laser, irradiation time, and laser power when photothermal effects of GNP are used for control release of drugs. The role of GNP as a radiosensitizer, a better drug delivery vehicle, and as an anticancer drug enhancer can be used effectively in the treatment of cancer by combining radiation therapy and chemotherapy for improved outcome in future cancer care. In addition, heat generating capability of gold nanostructures is also being used as a therapeutic technique as discussed in the next section.

## 4. Photothermal Therapy

Photothermal therapy (PTT) is less invasive compared to radiation therapy, chemotherapy or surgery and has drawn increased attention in cancer therapy. In PTT, optical radiation is absorbed and transformed into heat causing irreversible damage to the targeted tissue. Gold nanostructures have been used in this regard to enhance the absorption of light at specific wavelengths for heat conversion. For example, when they interact with resonant electromagnetic radiation, the coherent collective oscillation of electrons in the conduction band induces large surface electric fields which greatly enhance the radiative properties of GNPs [109]. This makes the absorption cross section of these NPs orders of magnitude stronger than that of the most strongly absorbing molecules [110]. The strongly absorbed radiation can be converted efficiently into heat due to electron-phonon and phonon-phonon processes and their potential use in PTT cannot be ignored. In addition, the interaction of light and gold nanostructures will depend on the type of laser used for the experiment. Currently, there are cw and pulsed lasers are available to irradiate the nanostructures with very different results. Ultrafast lasers will provide low average power therefore minimizing the tissue damage and very high instantaneous power that could result in high local temperatures. In PTT applications, the outcome is different based on the type of laser used. Terentyuk *et al.* used GNSs to produce a controllable laser hyperthermia in tissues with the aim of enhancing cancer PTT [111]. They have shown that both cw and pulse laser heating have similar short time kinetics (temperatures rise up to 46 to 50 °C over time, no more than 40 sec); however, pulsed laser heating seems more controllable than cw on a large time scale [111]. The gold-based PTT technique can be further improved through targeting of nanostructures as discussed in the next section.

The ability to target these nanostructures greatly reduces the required laser power for photothermal destruction of the targeted tissue and minimizes the damage to the surrounding healthy tissues. Recent studies have shown that GNPs could effectively damage the targeted tumor tissue when irradiated with wavelengths around their absorption peak [14,96,112-114]. In addition, the spectral tuning of NP resonance to the “therapeutic optical window” (750 to 1100 nm) can be achieved by variation in the particle size, shape, and structure [115-119]. Gold nanostructures can be synthesized with different sizes and shapes to tailor the absorption wavelength to NIR region for generation of heat. GNSs, GNRs, and GNCs are being used extensively in this regard and when irradiated with laser of suitable wavelength, these NPs could kill cancer cells [13,100,120]. However, spherical colloidal GNPs are not preferred in PTT since they absorb light in the visible range, which has a shallow penetration depth in tissue as compared to the therapeutic window in the NIR region where blood and soft tissue are relatively transparent. By exposing NPs to laser radiation near therapeutic optical window, it is possible to produce local heating of NP labeled cells without harming surrounding healthy tissues. Calculated absorption and scattering coefficients for spherical GNPs, GNRs, and GNSs are given in supplementary Table S2 [121]. In the next section, use of GNRs, GNSs, and GNCs for PTT is explained using recently published work.

**Figure 7.**
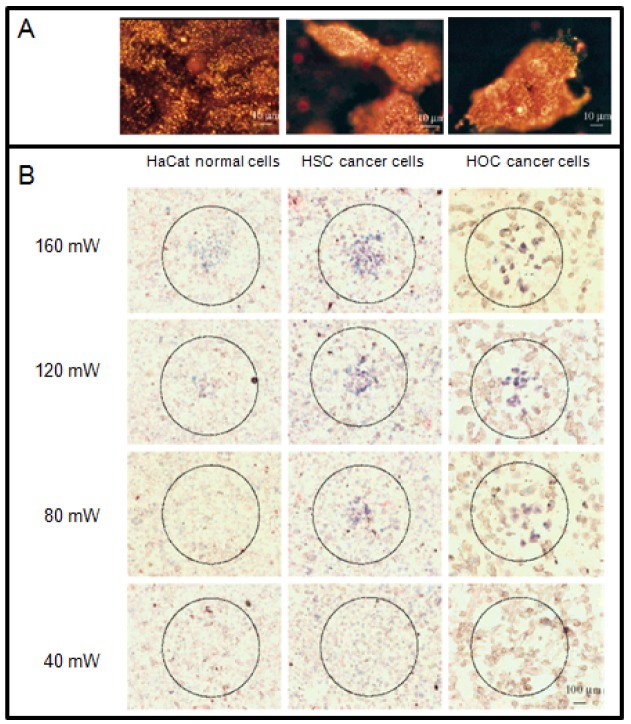
Gold nanorods (GNRs) for PTT. (**A**) Light scattering images of anti-EGFR/GNRs after incubation with cells (HaCat normal cell, HSC and HOC cancer cells) for 30 min at room temperature. (**B**) Selective PTT of cancer cells with anti-EGFR/GNRs incubated. The circles show the laser spots on the samples. At 80 mW (10 W/cm^2^), the HSC and HOC cancer cells are obviously injured while the HaCat normal cells are not affected. The HaCat normal cells start to be injured at 120 mW (15 W/cm^2^) and are obviously injured at 160 mW (20 W/cm^2^). Reproduced with permission [14].

Among other gold nanostructures, GNRs provide enough contrast for simultaneous molecular imaging and PTT due to their strong absorption and scattering in the visible and NIR region [14,96,122,123]. The appropriate aspect ratio of the GNR can be used to tune the NP absorption to the NIR region. By simply changing the dimensions of the GNR, the strong longitudinal plasmon absorption band can be tuned to various wavelengths, mostly to the NIR region where light penetration through tissue is optimal and minimal damage is expected to occur to the surrounding normal tissues [124]. Suitable aspect ratios of GNRs are usually from 3.8 to 6 [124]. El-sayed and coworkers have demonstrated *in vitro* that the GNRs can be used as novel contrast agents for both molecular imaging and PTT as illustrated in Figure 7 [14]. Functionalization of GNRs with antibodies allows their specific attachment to any target cell [125]. For example, anti-EGFR (epidermal growth factor receptor) antibody-conjugated GNRs bind specifically to the surface of the malignant type cells with a much higher affinity due to the over expressed EGFR on the cytoplasmic membrane of the cancer cells. As a result of the strongly scattered red light from GNRs, the cancer cells are clearly visualized and distinguished from the normal cells (Figure 7A). For the laser irradiation experiment, a cw Ti:sapphire laser at 800 nm was used. This wavelength is in the NIR region at which the tissue has low absorption. It also overlaps efficiently with the longitudinal absorption band of the GNRs. The cells were immersed in the anti-EGFR conjugated GNRs solution (Optical Density_800 nm_ = 0.5) for 30 min, rinsed with PBS buffer, and then exposed to the red laser light at power values of 40, 80, 120, 160, and 200 mW with a focus spot of 1 mm in diameter for 4 min each. The cells are then stained with 0.4% trypan blue for 10 min to test for their photothermal stability. Dead cells accumulated the dye and were stained blue, while living cells eliminated it and remained clear. Figure 7B shows images of samples irradiated at different laser energies. It was found that the two malignant cells require about half the energy needed to kill the nonmalignant cells, which is due to the over expression of receptor, EGFR, on the cancer cells and the corresponding higher amount of anti-EGFR antibody-conjugated GNRs which absorb the light and convert it into heat at the cell surface. The photothermally hot nanorods thus deliver more heat to the malignant cell membrane, leading to cell death at lower energy than that for the nonmalignant cells. The above results suggest that GNRs conjugated with anti-bodies can be used as a selective and efficient photothermal agent for PTT using a low-energy harmless NIR laser. Thus, for further *in vivo* applications, it is expected that the tumor tissue will be selectively destroyed at laser energies which will not harm the surrounding normal tissue due to the higher concentration of GNRs selectively bound to the tumor tissue.

**Figure 8.**
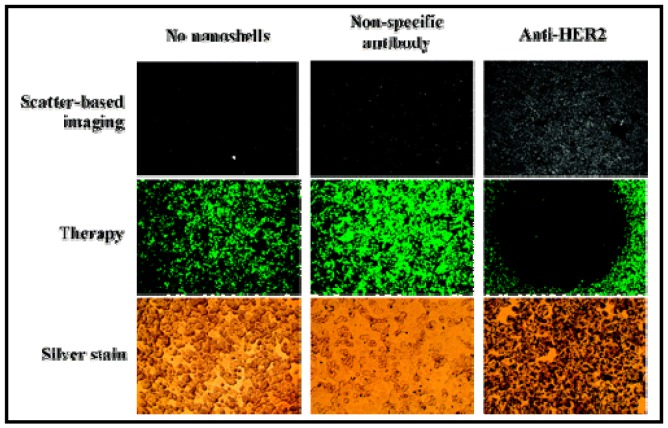
Use of gold nanoshells (GNSs) for PTT. Combined imaging and therapy of breast cancer cells using HER2-targeted GNSs. Scatter-based darkfield imaging of HER2 expression (top row), cell viability was tested via calcein staining (middle row), and silver stain assessment of GNS binding (bottom row). Reproduced with permission [13].

GNSs are another interesting type of gold-based NPs used in PTT. They are layered colloids with a nonconducting NP core covered by a thin gold shell [13,126,127]. By manipulating the thickness of the layers, these GNSs can be designed to absorb specific wavelengths of light and the most useful GNSs are those that absorb NIR light. West and coworkers have demonstrated how these GNSs can be engineered to both scatter light in the NIR enabling optical molecular cancer imaging and to absorb light for selective destruction of targeted cancer cells through PTT [13,126,127]. Figure 8 shows the capability of imaging using the laser scattering power of the GNSs. On exposure to 35 W/cm^2^ NIR light, human breast cancer cells incubated with GNSs *in vitro* undergo photothermally induced cell damage. Cells without GNSs display no loss in viability. Likewise, *in vivo* studies under magnetic resonance guidance reveal that solid tumors treated with GNSs and exposed to low-dose (4 W/cm^2^) NIR light incur a temperature increase of 37.4 °C within 4-6 minutes. The tissue displays coagulation, cell shrinkage, and loss of nuclear staining, indicating irreversible thermal damage. The tissue treated without GNSs attained significantly lower temperatures and appeared undamaged. The ability to control both wavelength-dependent scattering and absorption of GNSs offers the opportunity to design GNSs which provide, in a single NP, both diagnostic and therapeutic capabilities [13,128,129].

**Figure 9.**
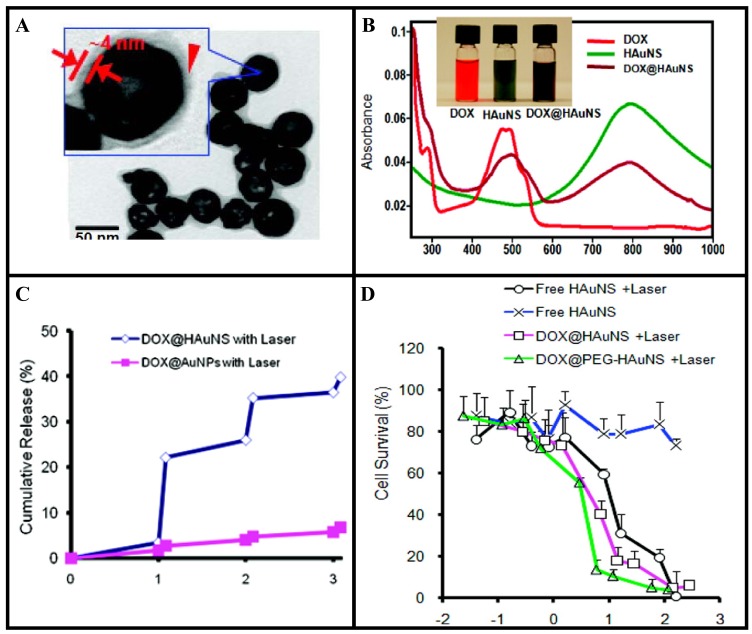
Hollow gold nanoshell (HGNS) mediated drug delivery and PTT. (**A**) TEM image of HGNS. (**B**) Absorbance spectra of free drug (DOX) (red), HGNS (green), and DOX@HGNS (brown). Inset: Photograph of aqueous solutions of free drug, HGNS, and drug@HGNS. Drug@HGNS displayed absorption peaks characteristic of both the drug and HGNS. (**C**) Release of drug from HGNS and GNP under repeated NIR laser exposure. (**D**) Cell survival as a function of drug concentration and gold concentration, respectively. Cells were either not exposed to NIR light or irradiated with NIR light (2 W/cm^2^ for 3 min per treatment, four treatments over 2 h). Reproduced with permission [130].

Hollow gold nanoshells (HGNSs) are a novel class of gold nanostructures having similar optical characteristics compared to GNSs [130-132]. However, HGNSs have more advantages due to their smaller size (30–50 nm in diameter) and the absence of a silica core (providing the opportunity to encapsulate drugs). Recent *in vivo* studies have shown that HGNSs have shown excellent colloidal stability, enhanced tumor uptake, and efficient PTT effect against human tumor xenografts after intravenous injection in mice [130,131]. Li and coworkers took one step further and demonstrated that HGNSs can be incorporated with anticancer drugs for combining chemotherapy and PTT [130]. TEM images and absorption spectra of HGNSs are shown in Figure 9A-B, respectively [130]. In this study by You *et al*, up to 63% of the anticancer drug, doxorubicin (DOX), by weight was loaded onto these NPs since the drug was coated on both the outer and the inner surfaces of the HGNSs. Irradiation with a NIR laser induced photothermal conversion, which triggered rapid drug release from drug-loaded HGNSs (Figure 9C). Significantly greater cell killing was observed when cells incubated with drug-loaded HGNSs were irradiated with NIR light which is attributable to both GNSs-mediated photothermal ablation and cytotoxicity of released free DOX (Figure 9D) [130]. The dual modality of cell killing using photothermal ablation mediated and controlled release of anticancer drugs upon NIR laser irradiation will significantly increase the likelihood of cell killing and potentially overcome resistance to chemotherapeutic agents, making this a promising approach towards improved cancer therapy.

**Figure 10.**
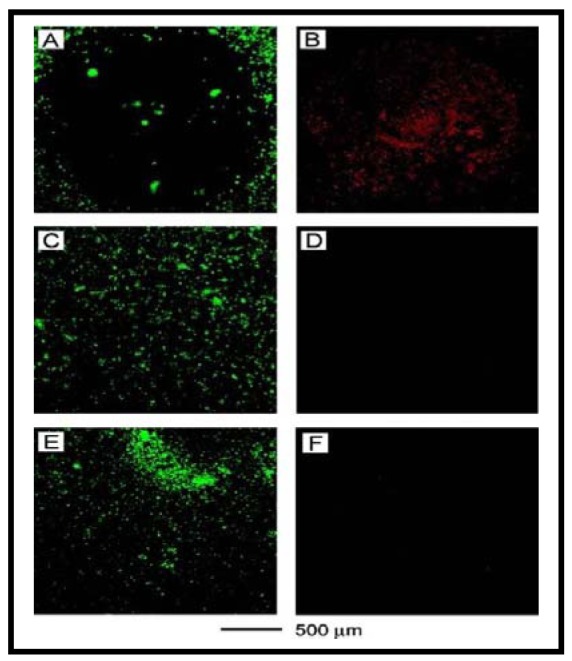
Gold nanocages (GNCs) for PTT. Cancer cells that were treated with GNCs and then irradiated showed a well-defined circular zone of dead cells as revealed by: (**A**) calcein AM assay (where green fluorescence indicates live cells), and (**B**) ethidium homodimer-1 (EthD-1) assay (where red fluorescence indicates dead cells). In the control experiment, cells irradiated under the same conditions but without GNCs treatment maintained viability, as indicated by (**C**) calcein fluorescence assay and (**D**) the lack of intracellular EthD-1 uptake. Cells treated with GNCs but irradiated at a lower power density remained alive, as shown by (**E**) calcein fluorescence assay and (**F**) the lack of intracellular EthD-1 uptake. Reproduced with permission [120].

So far, we have discussed the use of GNRs, GNSs, and HGNSs for PTT. Recently, GNCs have been introduced as a novel class of nanomaterials for PTT applications. As illustrated in Figure 10, Chen *et al.* used an optical spectroscopy based assay to further verify the damage caused in the presence of GNCs after laser irradiation [120]. The cells that were treated with immuno GNCs showed a well-defined circular zone of dead cells as revealed by: (A) calcein AM assay (where green fluorescence indicates the cells were live), and (B) ethidium homodimer-1 (EthD-1) assay (where red fluorescence indicates the cells were dead). Cells irradiated under the same conditions but without immuno GNC treatment maintained viability, as indicated by (C) calcein fluorescence assay and (D) the lack of intracellular EthD-1 uptake. Cells treated with immuno GNCs but irradiated at a lower power density (0.5 W/cm^2^) for 5 min remained alive, as shown by (E) calcein fluorescence assay and (F) the lack of intracellular EthD-1 uptake. GNCs are emerging as a versatile NP platform for drug delivery and PTT. A recent review article by Xia and coworkers highlights recent developments in the use of GNCs for drug delivery and PTT [100]. Interested readers are encouraged to read recent articles published on this topic for further information [20,100,108,120,135-137]. In addition, GNCs have also been explored as a contrast agent in optical coherence tomography, and photoacoustic tomography. So far, we have discussed the possibility of using gold nanostructures for radiation therapy, drug delivery and PTT. Recently, researchers have expressed interest in the use of gold nanostructures for PDT, as discussed in the next section.

## 5. Gold Nanostructures for Improved Photodynamic Therapy

Photodynamic therapy (PDT) is a promising technique that involves light, photosensitizers, and tissue oxygen [138]. A recent review by Juarranz *et al.* provides a detailed description of the activation mechanisms of the photosensitizing agent used in PDT [139]. Light-mediated activation of photosensitizing agents results in the generation of reactive oxygen species which destroys the target tissues [140]. The photosensitizers can be excited with light of an appropriate wavelength, and can generate highly reactive oxygen species inducing apoptosis or necrosis directly. Recently, several delivery strategies have been explored to overcome difficulties, such as less water solubility of drugs, by incorporating them into liposomes, polymeric micelles, conjugated polymer NPs, and colloidal silica-based NPs [137,141-143]. GNPs have also been identified as a promising delivery platform for PDT drugs [144]. NPs can be stabilized by steric repulsion to inhibit colloid aggregation in physiological conditions by using water-soluble polyethylene glycol (PEG) [145-147]. The PEGylated GNPs show remarkable resistance in protein adsorption and drugs on the NPs could be protected from being uptaken by the reticuloendothelial system (RES) [71,95,148,149]. GNP-based delivery systems could provide “enhanced permeability and retention” (EPR) effect where drugs can preferentially accumulate in tumor sites through the leaky tumor vasculature and minimize their return back into circulation [95].

**Figure 11.**
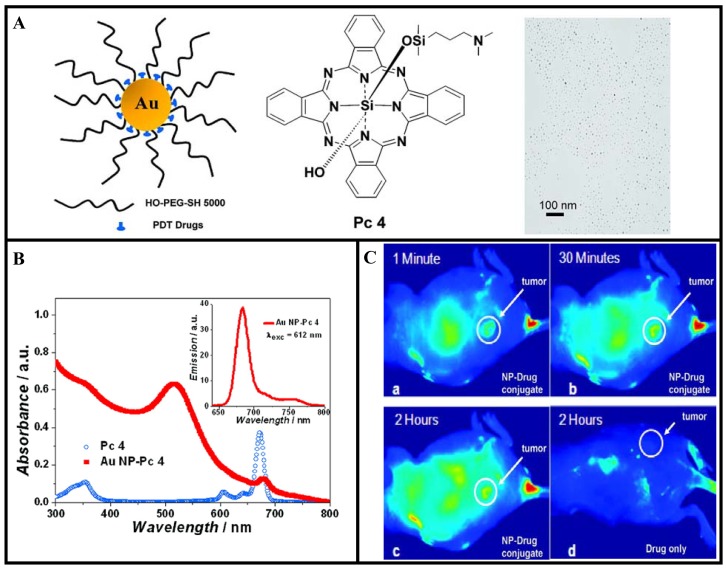
GNPs for PDT. **A**) Design of the water-soluble GNPs as a PDT drug delivery agent, structure of the PDT drug (Pc 4), and TEM image of the conjugates. **B**) Absorption and emission spectra (inset) of PEGylated GNP-drug conjugates. **C**) Fluorescence images of a tumor-bearing mouse after being injected with GNP-drug conjugates, (**a**) 1 min, (**b**) 30 min, and (**c**) 120 min after intravenous tail injection. Any bright signal is due to drug fluorescence, without which no fluorescence signals were detected from the mouse. Unprecedented delivery efficiency and accumulation rate of the drug in the tumor are monitored via the fluorescence increase in the tumor area (white circle). For comparison, a mouse that got only a drug formulation without the GNP vector injected is shown in panel (**d**). No circulation of the drug in the body or into the tumor was detectable 2 h after injection without the GNP as drug vector. Reproduced with permission [79].

Recently, Cheng *et al.* have used GNP vectors for *in vivo* delivery of PDT drugs [79]. They conjugated a PDT drug, silicon phthalocyanine 4 (Pc 4), onto the GNP as illustrated in Figure 11A. Silicon phthalocyanine 4 (Pc 4) is a hydrophobic PDT drug currently under Phase I clinical trials [150,151]. Figure 11B shows the UV-vis and fluorescence spectra of the drug conjugated GNPs in aqueous solution. The spectra demonstrate that the drug molecules adsorb on the PEGylated GNPs. When the GNP conjugated drug is injected for *in vivo* PDT, it usually takes 1 or 2 days until sufficient drug reaches the tumor site. The time for the maximum drug accumulation to the target tumor has been greatly reduced with the use of GNP-based drug conjugates compared to the free drug as illustrated in Figure 11C. The reported model system for PDT drug delivery is of unique versatility since it allows drug delivery, quantitative monitoring of the delivery process, and cancer therapy. However, more work needs to be done to optimize NP-based drug delivery vehicles for efficient tumor targeting. For example, the fluorescence images of tumor-bearing mouse showed localization of the drugs not only in the tumor but also in other areas as well (see Figure 11C). These types of model delivery systems can be further improved for active targeting of tumor by functionalizing them further with receptor-specific targeting ligands. Any future improvements of this PDT drug delivery process can thereby easily be monitored and quantified. In addition, GNPs can be incorporated into liposomes to use their heat generating ability to release liposome-entrapped drugs and pharmaceuticals upon irradiation by either UV or NIR light [152-154]. It may be noted that irradiated GNPs serve as energy collectors and work as localized heat sources by absorbing the energy of radiation. Therefore, these novel GNP-based delivery vehicles will play a bigger role in PTT.

## 6. Future Generation of Gold-Based Multifunctional NPs for Therapeutics and Imaging

The flexibility in engineering and construction of lipid or polymer-based vehicles makes them effective candidates for effective drug delivery and imaging [155-157]. GNPs have been incorporated into polymer or lipid based NP systems in order to build better multifunctional devices to facilitate imaging and drug delivery [80,158-160]. NP-based therapeutic systems that incorporate therapeutic agents, molecular targeting and diagnostic imaging capabilities are emerging as the next generation of multifunctional nanomedicine to improve the existing treatment options. In the treatment of cancer with radiation and chemotherapy, local tumor control can be improved when radiation is administered synchronously with the chemotherapeutic agent. However, incorporation of gold nanostructures into these existing clinical protocols will provide better opportunities since these NPs can be used to further enhance the damage caused by radiation and chemotherapy.

**Figure 12.**
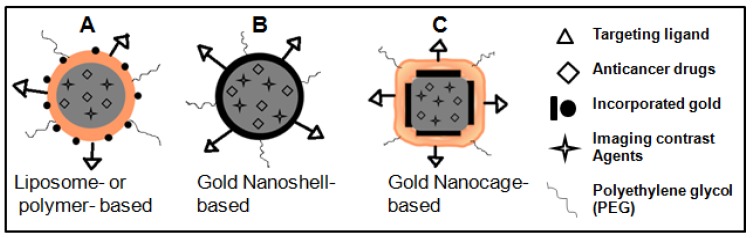
Future generation multifunctional NPs for improved therapeutics and imaging. Gold incorporated liposome or polymer NP, HGNS, and Polymer covered GNC as a new class of nanomaterials for improved therapeutics and imaging in cancer therapy.

As illustrated in Figure 12A, GNPs can be incorporated into the surface of the polymer or liposome NPs while keeping imaging contrast agents and anticancer drugs encapsulated in the core for combining imaging, radiation therapy, and chemotherapy for improved outcome in future cancer care [159]. Although not used in clinics until now, the dual modality of cell killing, using photothermal ablation mediated and control release of anticancer drugs by HGNSs and GNCs upon NIR laser irradiation, will significantly increase the likelihood of cell killing and potentially overcome resistance to chemotherapeutic agents, making it another promising approach to future cancer therapy (see Figures 12B–12C) [15].

## 7. Conclusions

Development of gold-based multifunctional NP-platforms offers great potential for introducing combinational therapy options for improvements in the care of cancer patients in the near future. The United States, Japan and the European Union have already established research initiatives to explore the potential medical applications of NPs. GNPs have also been shown to be non-toxic to human cells (U.S. National Cancer Institute-06-C-0167) [76]. Hence, the clinical perspectives of gold-based multifunctional NPs are promising. However, GNP-based platforms are still at the initial stage of development and much more research is required before they can be applied in clinical applications. It is also necessary to address safety and toxicological issues in order to capitalize on the full potential of NP-based therapeutics in cancer therapy. A close collaboration among those working in drug delivery and NP toxicology is very important in order to address these issues. Several articles have been published in order to address these issues and readers are encouraged to refer to them for detailed information [74,161-164].
